# First steps of laparoscopic surgery in Lubumbashi: problems encountered and preliminary results

**DOI:** 10.11604/pamj.2015.21.210.6689

**Published:** 2015-07-23

**Authors:** Willy Arung, Nathalie Dinganga, Emmanuel Ngoie, Etienne Odimba, Olivier Detry

**Affiliations:** 1 Department of General Surgery, University of Lubumbashi Clinics, University of Lubumbashi, Lubumbashi, Katanga Province, Democratic Republic of Congo; 2 Center of Research and Development in Surgery, University of Liège, Liège, Belgium, bAU2; 3 Department of Abdominal Surgery and Transplantation, University Hospital of Liège, University of Liège, Liège, Belgium

**Keywords:** Laparoscopy surgery, Lubumbashi, appendicetomy, adhesiolysis, cholecystecomy

## Abstract

For many reasons, laparoscopic surgery has been performed worldwide. Due to logistical constraints its first steps occurred in Lubumbashi only in 2008. The aim of this presentation was to report authors’ ten-month experience of laparoscopic surgery at Lubumbashi Don Bosco Missionary Hospital (LDBMH): problems encountered and preliminary results. The study was a transsectional descriptive work with a convenient sampling. It only took in account patients with abdominal surgical condition who consented to undergo laparoscopic surgery and when logistical constraints of the procedure were found. Independent variables were patients’ demographic parameters, staff, equipments and consumable. Dependent parameters included surgical abdominal diseases, intra-operative circumstances and postoperative short term mortality and morbidity. Between 1^st^April 2009 and 28^th^ February 2010, 75 patients underwent laparoscopic surgery at the LDBMH making 1.5% of all abdominal surgical activities performed at this institution. The most performed procedure was appendicectomy for acute appendicitis (64%) followed by exploratory laparoscopy for various abdominal chronic pain (9.3%), adhesiolysis for repeated periods of subacute intestinal obstruction in previously laparotomised patients (9.3%), laparoscopic cholecystectomy for post acute cholecystitis on gall stone (5.3%) and partial colectomy for symptomatic redundant sigmoid colon (2.7%). There were 4% of conversion to laparotomy. Laparoscopic surgery consumed more time than laparotomy, mostly when dealing with appendicitis. However, postoperatively, patients did quite well. There was no death in this series. Nursing care was minimal with early discharge. These results are encouraging to pursue laparoscopic surgery with DRC Government and NGO's supports.

## Introduction

For many decades now, laparoscopy surgery stands for minimal invasive surgical approach of the abdominal cavity. Its indications have been more and more extended. Since 1988, coelioscopic cholecystectomy has become a standard procedure of removal of gall bladder [1]. From 1992, gynaecologic surgical treatment has been carried out laparoscopically. Since this date, classical abdominal surgeries enter more and more in new laparoscopy registry [2]. While laparoscopic surgery is now performed routinely in all developed worlds, its first steps have hardly occurred in many developing countries like at our Lubumbashi school of Medicine, due to several constraints. We hereby acknowledge the contribution of the Don Bosco missionary hospital, (LDBMH) to the establishment of this new surgical abdomen approach. The missionary hospital has been providing university surgeons with training and practice of laparoscopic surgery, disposing of necessary equipment and consumable as well as Belgian cooperation trainers. The aim of this study was to report our ten-month experience of laparoscopic surgery at the LDBMH: problems encountered and preliminary results.

## Methods

### Study settings and staff training

Lubumbshi is the second big town of the Democratic Republic of Congo (DRC) administratively but also by its infrastructure and population of about three millions. With its copper, cobalt, uranium mines and other minerals, Lubumbashi was used to be called the “DRC economic capital” or the “DRC lung”. The world economic crisis has dramatically altered this situation but it is still being called Copper Capital”. The LDBMH is located near the Lubumbashi University administrative building along Kasapa road that leads to Lubumbashi University main campus. It is a multidisciplinary health center directed by the Don Bosco congregation established in the DRC for many decades. The institution enjoys support by the congregation but also the Belgian Government and many ONGs. The LDBMH utilises many general medical practitioners and several Lubumbashi medical specialists. A part from the supervisor and the surgeon trained abroad (the first in the department of visceral and digestive surgery, CHU Amiens-Nord (France) and the second in the department of digestive, endocrine surgery and transplantation, CHU of Liege, Belgium) the staff had two sessions of training in laparoscopic surgery in Lubumbashi by European Union ONG: “Doctors Without Holidays”. Nursing and paramedical staff included also four nurses: assistant, scrub, runner and technician nurses and two anesthetists. All were trained fort two months.

### Study time frame

The study was undertaken from 1^st^ April 2009 to 28^th^ February 2010.

### Study design, sampling and inclusion criteria

The study was a transsectional descriptive work with a convenient sampling. During the study time frame, the procedure was performed only to the patients with abdominal surgical conditions who consented and when logistical constraints of the procedure were sound: trained staff, fitness and availability of equipments and consumable. Independent variables were patients’ demographic parameters, appropriate staff, equipments and consumable. Dependent parameters included surgical abdominal diseases, intra-operative circumstances and postoperative short term outcomes (morbidity and mortality.)

### Data collection, data analysis

Pre-established forms were filled in with independent data (age, sex, disease, operating staff) and dependent variables (intra-operative events, postoperative mortality; morbidity, hospital stay and delay for discharge). Our data were introduced and analyzed using 2007 Excel and 2005 Epi info logiciels. Mean (average) and its parameters’ distribution were determined for Gauss distributions and median and its parameters’ distributions were measured for non-Gauss distributions; as well as standard deviations.

## Results

### Age and sex distribution

A total of 75 procedures were performed making an average of 7 cases per week, 2 cases per working day. During the study period the 75 laparoscopic surgeries accounted for 1.5% of the total abdomen activities at the LDBMH. Mean age was 30.25±2.02 years and median at 34,5years. Females were predominant with a sex ratio of 2F/1M, as it is shown on Table 1.

**Table 1:** Laparoscopic surgery at LDNMH, Patients’ sex distribution

Sex	Number	%
		
Males	27	36
Females	48	64
		
Total	75	100

### Causes and performed procedures

**Figure 1:**
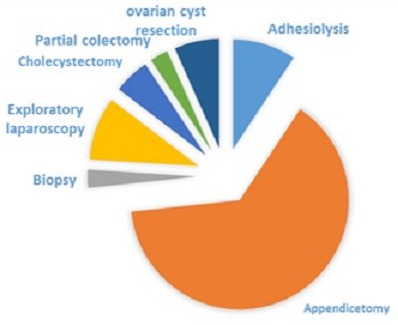
Laparoscopy surgery at ldbmh

As it is shown in Table 2 and diagram 1 the main perfor med procedure was appendicectomy (64%), followed by exploratory laparoscopy (9,3%), adhesiolysis (9,3%), cholecystectomy (5,3%) and other miscellaneous. The above mentioned causes are the commonest general surgical diseases in our environment (Figure 1).

**Table 2:** Procedures, indications/findings and frequency of laparoscopic surgery at LDBMH

	Indications:/Findings	Procedures	Frequency	%
				
Emergency laparoscopy	- Acute Appendicitis	Appendicectomy	48	64.0
	- Perforated stomach	Suturing+ lavage	2	2.7
	- Intestinal obstruction	Adhesiolysis	7	9.3
				
Elective laparoscopy	Post calculous cholecystitis	Cholecystectomy	4	5.3
	Symptomatic redundant sugmoid colon	Partial colectomy	2	2.7
	Chronic pain + /- mass			
	- Mesenteric adenopathy	Biopsy	1	1.3
	- Ovarian cyst	Excision	5	6.7
	- Liver mass	Biopsy	2	2.7
	- Gastric tumour	Lavage + closure	1	1,3
	- Normal findings	Lavage + closure	3	4.0

### Progression of laparoscopic surgery

**Figure 2:**
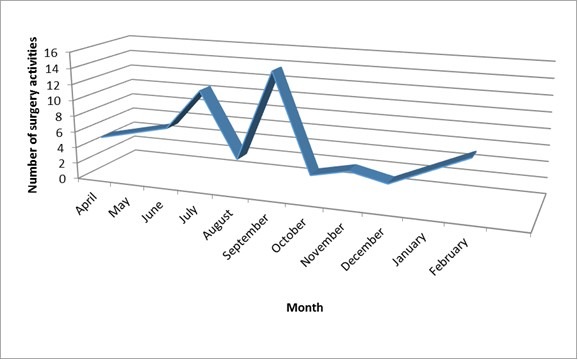
Laparoscopic surgery at ldbmh progress overview

**Figure 3:**
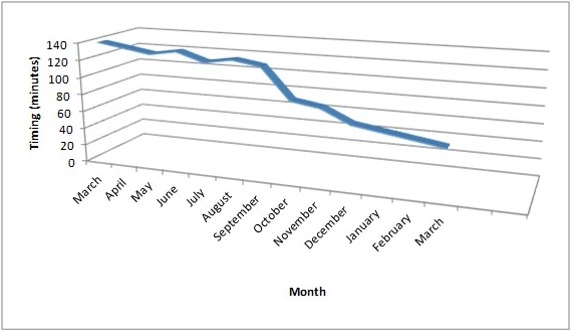
Laparoscopic surgery timing (minutes) overview on appendicectomy

As shown on diagram 2, the laparoscopic surgery activities increased at the beginning of the study time frame but decreased at the end of year 2009, period where only emergency surgeries were performed. There was again some increase around February 2010. Meanwhile there was improvement of the performance noted by the reduction of average duration of operations (diagram 3) following the acquisition of skills and mastering of events. It was also been noted by the low rate of conversion from laparoscopy to laparotomy and the reduction operation duration as it is shown on diagram 3 (appendicetomy), (Figure 2, Figure 3).

### Origin of patients and costs

**Figure 4:**
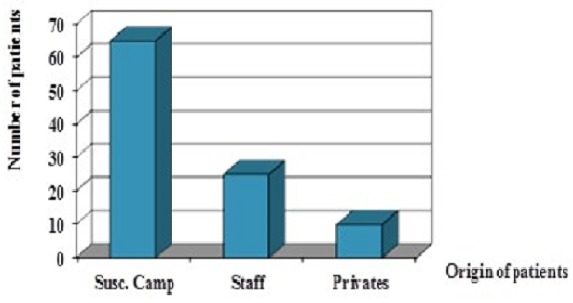
Origin of patients

As shown on Diagram 4, (Legend: Subscribed companies: Susc. Camp) private patients were rare. Patients mainly originated from subscribed companies that had schemes with the missionary hospital or from LDBMH staff. The laparoscopic surgery was and is still the surgery for few people who could pay directly or indirectly at LDBMH (Figure 4).

### Outcomes

There were 4% of conversion to laparotomy and there was no mortality in this series and all patients got discharged after a hospital stay relatively shorter than that after surgical laparotomy for each disease. For appendicectomy, postoperative pain was less severe lasted shorter after coelioscopy surgery. The average hospital stay after laparoscopic appendicectomy was three days while this average was of six days after open appendicectomy.

## Discussion

The adoption of laparoscopy in developing countries has been sporadic and minimal, despite the significant improvements in surgical care in developed countries. To uptake laparoscopic surgery in these countries, many authors have discribed some barriers [1–4]. Ian Choy [4] has reported three overarching barriers emerged: the organizational structure for funding laparoscopic procedures, the hierarchical nature of the local surgical culture, and the expertise and skills associated with a change in practice. The first barrier showed how the number of laparoscopic cases was limited by the ongoing funding structure, rather than upfront costs, of the laparoscopic program. The description of the second barrier showed the importance of understanding the local surgical culture in attempts to adopt new technology. The third barrier emphasized the fact that due to the generalist nature of surgical practice, surgeons were less willing to practice more technically complicated procedures [4]. Due to logistical constraints the first steps of laparoscopic Surgery occurred in Lubumbashi only in 2008, at the Don Bosco missionary hospital, institution that provided university surgeons with training and practice of this minimal invasive abdominal approach. By the way, in our environment, laparoscopic surgery still appears as a very costful technique, the surgery for minority either very rich citizen or privileged patients from LDBMH staff and subscribed companies with the hospital. It requires, indeed, special costful equipment and consumable as well as high skilled staff. This situation is similar in most of Africa country: The number of surgeons per capita in many African countries is low [5]. Migration of health care providers - whether to the private sector, to nongovernmental organizations (NGOs), or overseas - places a strain on the ability of a country to provide essential surgical services [6]. Adequate facilities are crucial to the provision of basic surgical care. Even the best trained and motivated surgical team cannot function without appropriate infrastructure. In Malawi, a study of district hospital theaters showed that most did not have dedicated theater staff, and half did not have adequate instruments to perform common operations. More than half did not have basic skin and soft tissue sutures available on the day of inspection [7].

In the international literature [3] and in Lubumbashi [8], appendicectomy has been the most performed procedures but cholecystectomy is the most laparoscopic surgery frequently performed [1]. However, the advantage of laparoscopic appendicectomy over the classical open appendicectomy is still controversial despite several randomized studies. The duration of the coelioscopic appendicetomy is longer than that of open appendicetomy. Surgical site infection is more frequent after open appendicectomy but there are more deep sepsis after coelioscopy than after open appendicectomy mainly in more complicated appendicitis. Postoperative pain is less severe and activity resumption is faster after coelioscopic appendicetomy. In this latter moreover the hospital stay is shorter. It has been reported that coelioscopic appendicectomy avoids unnecessary appendicectomy especially in sexually active women and that it has more other advantages than open appendicetomy in young women, in patients with professional activity and in obeses. However other authors [9] have reported negative opinion and mentioned that clinical advantages of coelioscopic appendicetomy are not substantial and that the risk of deep abscess has to be taken in account, especially in perforated or gangrenous appendicitis. However coelioscopic advantages on postoperative comfort, wound healing, early discharge from hospital, fast duty resumption, lesser after postoperative intra-abdominal adhesions that use to cause intestinal obstruction, chronic pain and infertility [10–13] pledge for its wider use in our environment also. For example, in USA [14], it has been illustrated that post-laparotomy adhesions had drastically been increasing operative time table lists as about three thousands adhesiolysis had to be carried out annually costing about 1,3 milliards US$.

## Conclusion

Laparoscopic surgery has its first steps at the Don Bosco Missionary Hospital. A substantial amount of patients with various intra-abdominal conditions have undergone this minimal invasive procedure with very fair outcomes: minimal wound infection, minimal postoperative pain, hospital short stay before discharge and without postoperative early manifestations of intra-abdominal adhesions. Two problems were mainly encountered. Only LDBMH staff, subscribed companies staff and few private patients could be operated because of the cost of equipments and consumable. The second was the duration of the procedure related to staff's skills and adaptability. This led to erratic recruitment with a low rate of the procedure with regard to all hospital abdominal surgical activities. With the time and as the staff got more experience, the time of the procedure was gradually reduced.

**Recommendation**: Laparoscopic surgery has led to better outcomes at the Lubumbashi Don Bosco Missionary hospital in all postoperative aspects. It is still restricted to few citizen due to its logistical and cost-effective requirements. Because of gotten encouraging results we recommend the following: the continuous medical education on this surgical subspeciality; the establishment of well equipped and functional laparoscopic surgery units at our Lubumbashi university hospitals and main country surgical departments
